# New combined experimental and DFT studies for adsorption of sole Azo-dye or binary cationic dyes from aqueous solution

**DOI:** 10.1038/s41598-024-65649-2

**Published:** 2024-06-26

**Authors:** Shaimaa M. Ibrahim, Nouf F. Al-Harby, Sahar. A. El-Molla, EL-Shimaa Ibrahim

**Affiliations:** 1 Department of Chemistry, Faculty of Education, Ain Shams University, Roxy, Cairo, 11711 Egypt; 2 Department of Chemistry, College of Science, Qassim University, 51452 Buraidah, Saudi Arabia

**Keywords:** Adsorption, DFT studies, Al_2_O_3_, Maxilon blue dye, Mechanism, Catalysis, Environmental chemistry, Theoretical chemistry

## Abstract

Textile-toxic synthetic dyes, which possess complex aromatic structures, are emitted into wastewater from various branches. To address this issue, the adsorption process was applied as an attractive method for the removal of dye contaminants from water in this article. An unprecedented integrated experimental study has been carried out, accompanied by theoretical simulations at the DFT-B3LYP/6-31G (d,P) level of theory to investigate how single Maxilon Blue GRL (**MxB**) dye or and its mixture with MG (Malachite Green) dyes interact with the adsorbent and compare the obtained results with the data obtained through experimentation. The full geometry optimization revealed the physical adsorption of dyes on the Al_2_O_3_ surface. Non-linear optical properties (NLO) results emphasized that the complex MG-Al_2_O_3_-MxB is a highly promising material in photo-applications, and the adsorbed binary system is energetically more favorable compared to the adsorbed sole dye system. The experimental results for (**MxB**) dye adsorption onto γ-Al_2_O_3_ affirmed that the optimum conditions to get more than 98% uptake were at dye concentration 100 ppm, pH 10, adsorbent content 0.05 g, and equilibrium time only 20 min. The kinetic and isothermal studies revealed that the adsorption accepted with the pseudo-second-order and Freundlich isotherm model, respectively. The removal efficiency of the mixture of **MxB** and **MG** dyes was the highest but did not change clearly with increasing the % of any of them. The details of the interaction mechanisms of the sole and binary dyes were proven.

## Introduction

Undoubtedly, today's environmental pollutants, specifically water pollution, are deemed huge obstacles mankind confronts. Water pollution necessitates a primary and inherent resolution due to its status as one of the most severe ecological predicaments. As an additional illustration, the textile industry generates a substantial quantity of effluent, which comprises numerous undesirable substances, such as corrosive dissolved solids, acidic and poisonous organic or inorganic compounds. A great number of dyes are hazardous and toxic to organisms, and they have the potential to induce a range of illnesses, including those suspected to be carcinogenic and mutagenic impact^1,2^. Various industrial sectors, particularly the dye manufacturing and textile finishing branches, release these substances into wastewater^3^. The textile synthetic dyes are derived not only from the food coloring, cosmetics, paper, and carpet industries but also from a variety of complex aromatic structures. These dyes possess intricate physico-chemical, thermal, and optical properties^4,5^. Azo dyes, for instance (**MxB**) has one or more nitrogen to nitrogen double bonds [–N=N–) and constitute a significant portion of dyes that are used in the textile industry^6–8^. Malachite green (**MG**) is a cationic dye pertinence to triphenylmethane group and its shining green color makes it appropriate for dyeing in different industries^9,10^. There are many ways to get rid of excess textile dye from water, one of this ways is the adsorption, reverse osmosis, and ultra-filtration, etc.^11–17^. Among others, the sorption process provides an attractive alternative for the treatment of contaminated water, especially if the sorbent is inexpensive and does not require an additional pretreatment step (such as activation) before its application^4,18^. Moreover, the utilization of this procedure, wherein the adsorbent content is kept at a minimum, becomes imaginable when dealing with diluted concentrations of contaminants in water. However, a complication arises when attempting to address the issue of water pollution caused by the presence of multiple dye types. Up until now, various academic institutions have endeavored to engineer novel adsorbents or modify existing ones in order to effectively remove a combination of cationic and/or anionic dyes, acting as organic pollutants, from the contaminated water^17–20^. Nevertheless, a lot of these adsorbents have several disadvantages in terms of high cost and difficulty of their preparation, so, in this article, the authors try to examine the adsorption process for a mixture of dyes over a cheap and easily prepare among all adsorbents as alumina. Alumina with numerous structural phases, namely α, β, γ, η, θ, κ, and χ is a well-known metal oxide, one of the special functional materials and common adsorbents used in environmental engineering and in a wide range of applications, including the electronics, metallurgy, optoelectronics, catalyst carrier, and fine ceramics, owing to its high purity, tiny particle size, uniform distribution, and high surface area^21,22^. On the other hand, compared to other powders, alumina powder is more pH sensitive since it is an amphoteric oxide. As a result, it's critical to keep the pH level and solution concentration consistent throughout the reaction solution. Recently, many studies about alumina have been conducted to examine its adsorption ability to remove pollutants as single cationic or anionic dyes from wastewater^21,22^. The majority of these investigations have mostly concentrated on increasing the dye's ability to be adsorbed on the adsorbent surface. Conversely, limited studies have been proceeded on the adsorption of mixtures of organic dyes on the alumina material. Further, neither experimental nor theoretical studies have been performed on alumina as an adsorbent for the adsorption of single **MxB** or its mixture with other cationic dyes from aqueous solution. Moreover, there is a lack of a thorough knowledge of the behavior of the interactions between the alumina adsorbent and the single dye, such as **MxB** or binary cationic dyes, in comparison to the experimental adsorption data. Herein, aiming to overcome these shortages, a great deal of information about the adsorption process and the interactions involved may be gained from quantum chemical simulations. Additionally, density functional calculations will be used to evaluate the plausibility of the adsorption of mixed dyes; geometrical features, electronic structures, and the adsorption properties of the interacting systems have all been thoroughly investigated. To bridge gaps from earlier studies and enhance the understanding of adsorption mechanisms, this work will be use the density functional theory in conjunction with the classical isotherm and kinetic models to provide a deeper description of the adsorption mechanism of single and binary dye molecules onto the solid adsorbent. Finally, it can be concluded that (DFT) theory has turned into a powerful and missionary tool to: (i) achieve the properties of a “functionalized” material to remove mixtures of cationic dyes; (ii) foresee the feasibility of adsorption of a particular adsorbent targeting a certain adsorbate and understanding of these molecular interactions^23^; and (iii) Investigate the nonlinear- optical properties of the resulted complexes after adsorption process. To the best of our knowledge, there is no report on the NLO properties of the complex **MG**-Al_2_O_3-_**MxB**.

The novelty of this paperwork will be appeared in evaluating the simulation study by (DFT) calculations for the behavior of the interactions between single or binary cationic dyes and the (γ-Al_2_O_3_) nanoparticles adsorbent against the experimental adsorption data. The Global reactivity Descriptors and NLO properties will be investigated theoretically. The detailed reaction mechanism for the dual system is going to be postulated. Experimentally, different parameters to determine the optimum condition toward (**MxB**) adsorption, like dye concentration, adsorbent content, pH, contact time, the kinetic and isotherm models will be studied.

## Experimental study

### Materials

Malachite Green (molecular formula C_52_H_54_N_4_O_12_; molecular weight: 927.02 g/mol and λ_max_ = 617 nm) and Maxilon Blue GRL (molecular formula C_20_H_26_N_4_O_6_S_2_; molecular weight: 482.57 g/mol and λ_max_ = 609 nm) were purchased from DyStar. Al (NO_3_)_3_ and analytical grade (NH_4_)_2_CO_3_ were purchased from (Merck) and (Win Lab), respectively, and distilled water was used throughout the studies.

### Preparation of adsorbent

The sample of alumina was prepared by the precipitation method and it is worth mentioning that the applied adsorbent in this work has already been reported previously, evaluated using X-ray diffractometer, the surface area (S_BET_), and pore volume measurements^24^. Briefly, a solution of aluminum nitrate was prepared, and NH_4_CO_3_ (1M) was added slowly to the solution with stirring until a precipitate was composed at pH 7 with stirring for one hour. Then, the precipitate filtration and drying were performed at 120 °C. Finally, the solid was calcined at 500 °C for 3 h and was nominated as Al-2.

### Characterization of adsorbent

For an additional estimate, the morphology of the adsorbent and its nanostructure are characterized by TEM (Jeol 2100).

### Adsorption studies

#### Single dye

Adsorption study of **MxB** on Al-2 was carried out in batch mode, where a fixed amount of 100 mg of (Al-2) adsorbent was inserted into 100 mL of the dye solution (initial concentration: 100 mg/L). The stirring of the obtained dye solution continued at the wanted temperature and pH . At different time intervals, the solution was taken out, centrifuged for 15 min (at 7000 rpm), and the absorbance was specified by a UV–Vis spectrophotometer (Jasco V-550, Japan) at λ ranging from 400 to 800 nm, with the maximum value obtained at 609 nm. The percentage of **MxB** dye removal and its adsorbed amount per unit adsorbent (mg/g) were calculated by applying Eqs. (1) and (2), respectively:

1 $$(\%)\text{ R }(\mathbf{M}\mathbf{x}\mathbf{B}) =\frac{Co - Ce}{Co} \times 100$$

2 $${q}_{e} = \frac{Co - Ce}{m} \times V$$

where V is the solution volume (L), C_o_ is the initial concentration (mg/L) of the dye, C_e_ is the equilibrium concentration of the dye in solution (mg/L), m is the weight of ɤ-alumina (g), and q_e_ is the adsorption capacity (mg/g). Then, studying the effect of different factors that have an influence on the removal percentage of **MxB** was done. The first one is the impact of pH ranging between 4 and 10, with an adsorbent dose of 0.1 g at room temperature, an initial **MxB** dye concentration 100 mg/L, and contact time (0–180). The second factor is the influence of the initial **MxB** dye concentration (10, 25, 50, 75, and 100 ppm) performed with an adsorbent dose 0.1 g at room temperature, pH 10, and at equilibrium time (20 min). Further, studying the impact of adsorbent doses (0.03, 0.05, and 0.1 g for 100 mg/L **MxB** dye solution, at pH 10, room temperature, and equilibrium contact time (20 min). Finally, the kinetics of **MxB** adsorption and the adsorption isotherms were investigated.

#### Mixed dyes

An adsorption study of binary **MxB** and **MG** dyes on Al-2 was conducted in batch mode under the following conditions: initial concentration (50 ppm), pH 7, and a fixed amount of 0.1 g of adsorbent. The adsorption procedure was consistent with what was previously described in the section on single-dye adsorption. Additionally, different ratios of **(1MxB: 1MG), (1MG: 2MxB)**, and **(2MG: 1MxB)** were examined. The percentage of removal and adsorption capacity for the various mixed dye ratios were calculated using Eqs. (1) and (2), respectively.

## Results and discussion

### DFT theoretical study for adsorption of single MxB and mixture of two cationic (MxB + MG) dyes

#### Ground state and geometrical parameters

**Figure 1:**
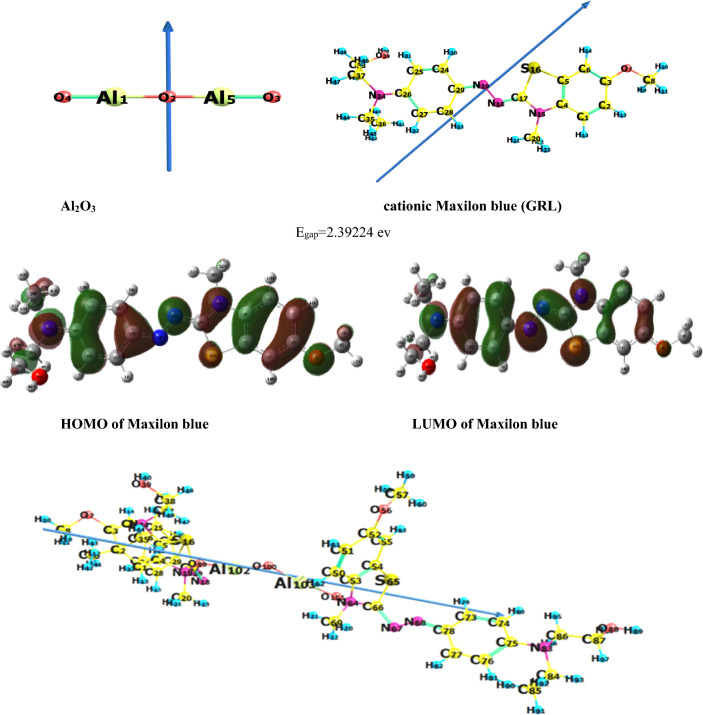
Optimized structure, numbering system, vector of dipole moment of Al_2_O_3_, **MxB** and **MxB**-Al_2_O_3_, HOMO, LUMO maps and energy gap of **MxB** using B3LYP 6-31G (d,p) level of theory.

Firstly, in this part, theoretical simulation of **MxB** adsorption on alumina as an individual cationic dye was done, followed by studying the theoretical adsorption of **MxB** and **MG** as a mixture of two cationic dyes in a binary system to get the most stable configuration of the dyes on alumina. The single system of **MG** adsorption alone on alumina was studied earlier^24^. Once the determination of the configurations of **MxB** and **MG** on Al_2_O_3_ was achieved, the calculation of the adsorption energy of these dye molecules on the adsorbent was performed. Additionally, the calculation of the dipole moment, which serves as an estimation of the molecule's polarity and reflects the extent of charge distribution within the molecular system, was performed. To analyze the structural geometry of the sole **MxB** cationic dye in the gas phase, a complete geometry optimization was conducted. This optimization encompassed the optimized bond lengths, bond angles, and natural charges, employing the B3LYP/6-31G (d, p) method, as presented in Table S1 "see supplementary data". The geometrical optimization parameters and the natural charges of single **MxB-Al**_**2**_**O**_**3**_ were inserted in Table S2 "see supplementary data". The obtained results revealed that the optimized structure for a single **MxB** dye adsorbed on alumina is planar. Further, Table 1 includes the measured values of total energy, E_HOMO_ (highest occupied molecular orbital energy), E_LUMO_ (lowest unoccupied molecular orbital energy), energy gap, and dipole moment for the reacting **MxB** and/or **MG** dyes with Al_2_O_3_, according to the frontier molecular orbital (FMO) theory of chemical reactivity. This table showed that the Al_2_O_3_ adsorbent revealed a high tendency to donate electrons to an appropriate acceptor molecule with low energy or an empty electron orbital, in our case as a single **MxB** or mixed with **MG** dye. Fig. 1 illustrates the HOMO and LUMO of **MxB** orbitals. The energy of the LUMO indicates the susceptibility of molecules to nucleophilic attack. Density Functional Theory has become a successful way to obtain a better understanding of chemical reactivity and stability about molecules, the HOMO-LUMO energy gap (c.f. Table 1 and Fig. 1) refer to the difference between the HOMO and LUMO energy values, which indicated that the charge transfer interaction taking place within the molecule (**MxB-Al**_**2**_**O**_**3**_) was lower (means at the end of the adsorption process), but it had the highest value at the initial time for the adsorbent and adsorbate molecules.

**Table 1:** Total energy, energy of HOMO, LUMO, energy gap, and natural charge of **MxB**, Al_2_O_3_ and **MxB**-Al_2_O_3_ computed at B3LYP/6-31G (d,P) level of theory.

Compounds	Maxilon blue (MxB)	Al_2_O_3_	MxB-Al_2_O_3_
E_T_ (au)	− 1505.31	− 710.60	− 3717.68
E_HOMO_ (eV)	− 8.19	− 7.55	− 2.79
E_LUMO_ (eV)	− 5.79	− 3.59	− 2.19
E_gap_ (eV)	2.39	3.95	0.59
B.E (a.u.) = E_**MxB-Al2O3**_ − (E_**MxB**_ + E_**Al2O3**_(= − 3717.67813972 – (− 1505.306*2 + − 710.599) = 3.53			
µ (Debye)	4.57	0	18.43
Ionization potential (I = − E_HOMO_) eV	8.19	7.55	2.79
Electron affinity (A = − E_LUMO_) eV	5.79	3.59	2.19
Electronegativity χ = (I + A)/2) eV	6.99	5.57	2.49
Chemical potential p = (− χ) eV^−1^	− 6.99	− 5.57	− 2.49
Chemical hardness (η = (I − A)/2) ev	1.20	1.98	0.30
Chemical softness (S = 1/2η) eV^−1^	0.42	0.25	1.68
Electrophilicity index (ω = p^2^/2η) eV	20.43	7.85	10.43

#### Global reactivity descriptors

At the B3LYP/6-31G (d, p) level of theory**,** electron affinity (**A, eV**), ionization potential (**I, eV**), chemical hardness (${\varvec{\eta}}$, eV), the chemical potential (***V***, eV^−1^), electronegativity (${\varvec{\chi}}$, eV), global softness (S, eV^−1^) and global electrophilicity index, (***ω***, eV) of individual **(MxB), Al**_**2**_**O**_**3**_**,** and **MxB-Al**_**2**_**O**_**3**_ were computed and mentioned in Table 1. In general, molecules characterized by a minimal energy difference are ascribed to promoting chemical reactivity, lowering kinetic stability and are also known as soft molecules; on the other hand, those with a large energy gap have higher stability and are looked as hard molecules because they resist charge transfer and changes in their electron density and distribution. The energy of HOMO is directly connected to the ionization potential (IP = − E*HOMO*), but the energy of LUMO is attached with the electron affinity (EA = − E*LUMO*). Additionally, by utilizing these values, attractive properties such as electronegativity (χ), chemical hardness (η), electrophilicity index (ω), and electronic chemical potential (V) can be given. It is known that the difference in electronegativity between adsorbent (alumina) and adsorbate (**MxB** dye) reflected a stronger aggressiveness of nucleophilic attack that can promote adsorption capacity (as shown in Table 1).

#### Non-linear optical properties (NLO)

As no experimental or theoretical investigation has been conducted to examine the nonlinear optical properties of these types of dyes, our research interest is directed towards undertaking this study. Due to its importance in providing crucial optical modulation, switching, laser, frequency shifting, fiber, optical materials logic, and optical memory for new technologies such as telecommunications, optical interconnections, and signal processing, NLO is at the forefront of current research^25,26^. In order to investigate the relation between molecular structure and NLO, the polarizabilities and hyperpolarizabilities of the studied **MxB** cationic dye, Al_2_O_3_, **MxB-**Al_2_O_3_, and the binary system complex are calculated using the B3LYP/6-31G(d,p) level of theory**.** The mean polarizability α, the anisotropy of the polarizability Δα and the mean first-order hyperpolarizability (β) were mentioned in Table 2. The polarizabilities and first- order hyperpolarizabilities are called in atomic units (a.u.), the calculated values have been transformed into electrostatic units (esu) using a conversion factor of 0.1482 × 10^–34^ esu^27^ for α and 8.6393 × 10^–33^ esu^28^ for β. In the NLO research, the standard prototype P-nitro aniline (PNA) is used. As there were no experimental values for the NLO characteristics of the molecules under research, PNA was used in this work as a reference. The magnitude of the molecular hyperpolarizability β is one of the key factors in NLO system. The analysis of the β parameter for the studied molecules showed that **MxB** is 61 times greater than PNA, the complex **MxB-**Al_2_O_3_ is 125 times greater than PNA, and the complex **MG**-Al_2_O_3_-**MxB** is 534 times greater than PNA, implying their hopeful applications as NLO materials. The variation of Hyper-Rayleigh scattering (β _HRS_) and the depolarization ratio (DR) appearing in Table 2 can be rationalized by complexation and structural evidence^29^. The high value of β and DR in the **MxB** form and the lowest value in the case of Al_2_O_3_ (**c.f. **Table 2) confirmed the long bond length between the N-atom of **MxB** and the O-atom of Al-oxide ^30^ and hence weak bonding, resulting in a physisorption process.

**Table 2:** The total mean polarizability (<**α**˃), the anisotropy of the polarizability (**Δα**), and the mean first-order hyperpolarizability (<**β**˃), of cationic **MxB**, Al_2_O_3,_ **MxB**-Al_2_O_3_ and **MG**-Al_2_O_3_-**MxB** computed at B3LYP/6-31G(d,P).

Property	PNA	MxB	Al_2_O_3_	MxB-Al_2_O_3_	MG-Al_2_O_3_-MxB
**<α > ** × 10^−24^esu	22	− 11.79	− 7.29	− 47.20	− 50.23
**Δα** × 10^−24^esu		28.58	6.77	20.94	9.01
<**β**>× 10^−24^esu^c^	15.5	948.52	0	1943.22	8282.07
DR		0.16	0	0.07	0.004
**β**_HRS_		41.06	0	174.87	74.03

### Theoretical study for the interaction of cationic dyes with Al_2_O_3_

#### Maxilon Blue (GRL)

From the theoretical study, the difference in the electronegativity and the other calculated parameters, we can predict the theoretical interaction between **MxB** and alumina. This interaction has been attributed to electrostatic forces between the cationic ion groups (–N^+^) of **MxB** and the negatively charged ion groups (–Al–O^−^) on the alumina surface. As the **MxB** molecule possesses an N atom that is positively charged, which facilitates the adsorption on Al_2_O_3_ surface. It is due to the lone pair on the N atom being delocalized over the ring, which makes the electrons less available and hence more subjected to attack by nucleophiles. The outcome is a creation of an electrostatic interaction between a positively charged N atom of **MxB** and a negative O atom in alumina. In the present study, two molecules of the cationic **MxB** and one molecule of Al_2_O_3_ have been used to form **MxB**-Al_2_O_3_ complex. The studied **MxB**, Al_2_O_3_ and **MxB**-Al_2_O_3_ complex were optimized at the B3LYP/6-31G (d, p) level of theory. The optimized bond length N–O between **MxB** (N) and the Al-oxide (O) was 1.37829 Å (literature experimental N–O is 1.1–1.36 Å^31^) "Table S2". The intensity of the contact between the **MxB** and Al surface decreases as the N–O bond increases, and as a result, the adsorption is physical. The optimum bond length between dye and oxide is larger than the experimental one, as revealed by the theoretical calculation, which supports the physisorption process.

#### Binary maxilon blue (GRL) and malachite green

**Figure 2:**
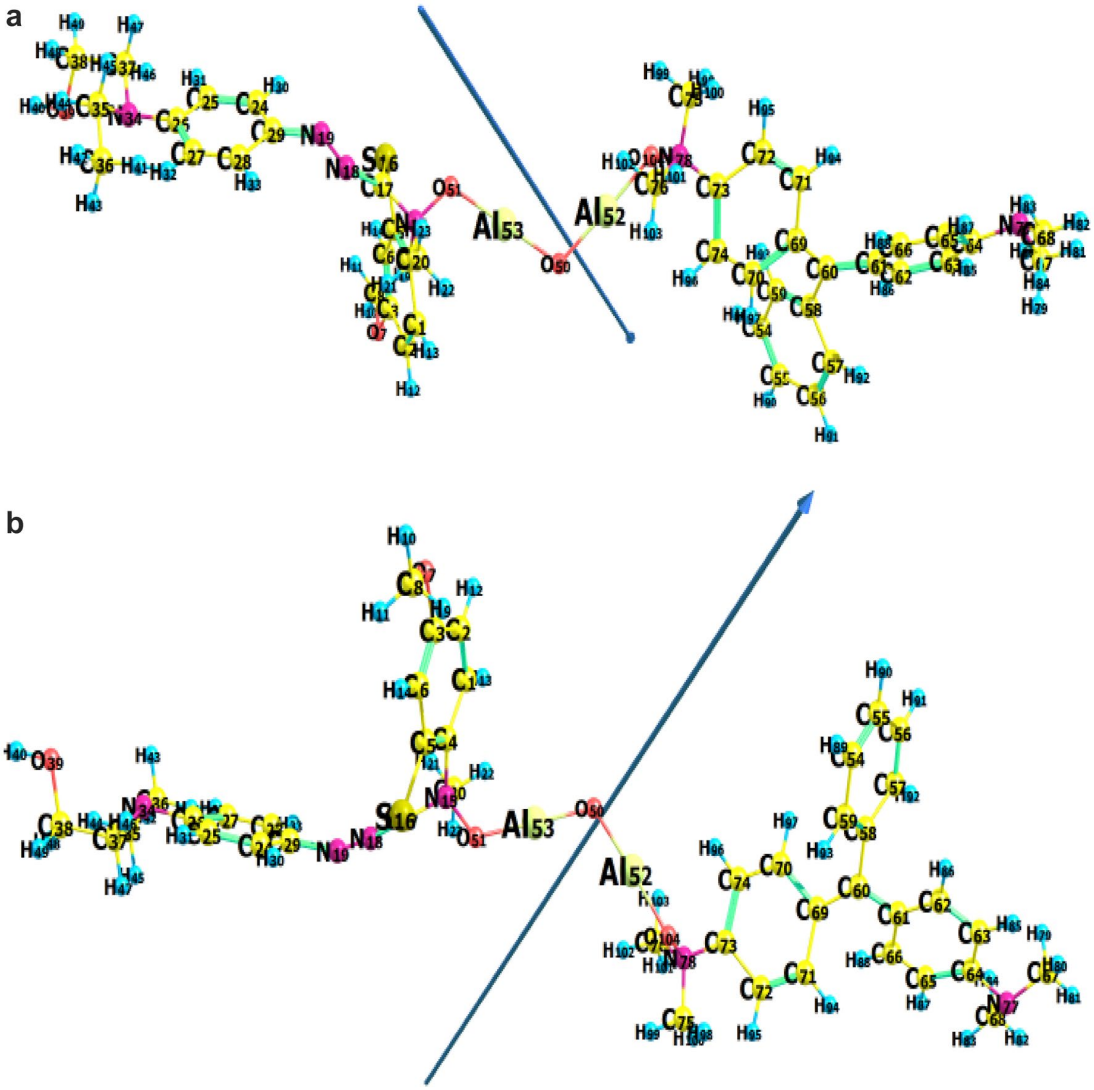
(**a**) Optimized structure, numbering system, vector of dipole moment, HOMO, LUMO maps and energy gap of **MG**-Al_2_O_3_-**MxB** calculating at B3LYP 6-31G (d,p) level of theory. (**b**) Optimized structure, numbering system and vector of dipole moment of **MxB-**Al_2_O_3_**-MG.**

The studied **MG**-Al_2_O_3_**-MxB** complex was optimized at the B3LYP/6-31G (d, p) level of theory. The optimized bond lengths, bond angles, dihedral angles, and the vector of the dipole moment are presented in Fig. 2 and Table S3 "see supplementary data". It was clear from the obtained results of dihedral angles that the optimized structure of the binary adsorbed system was non-planar. Also, the total energy, energy of HOMO and LUMO, energy gap, binding energy (a.u.), dipole moment, the ionization potential (I, eV), electron affinity (A, eV), chemical hardness (η, eV), global softness (S, eV^−1^), chemical potential (V, eV^−1^), electronegativity (χ, eV), and global electrophilicity index, (ω, eV), of **MG-**Al_2_O_3_**-MxB** were estimated and listed in Table 3. Many theoretical reactivity descriptor parameters, such as E_HOMO_, E_LUMO_, energy gap, global softness, and dipole moment, are used in discussing the mechanism of chemical reactions. It is known that the molecule E_HOMO_ reflects its electron-donating ability, while the E_LUMO_ represents its electron-accepting ability^32^. In Tables 1 and 3, it is clear that the E_HOMO_, E_LUMO_, energy gap, electronegativity, ionization potential, and chemical softness values for the two cationic **MxB** and **MG** dyes are relatively close together. So, these data referred that there is no preferable dye in the mixed binary system for adsorbing over alumina. Further, the dipole moment μ (Debye) and the electrophilicity ω (eV) reflect the polarity of the whole molecule, so higher the dipole moment, the electrophilicity values, the high chemical reactivity will be obtained. Tables 1 and 3 showed that the sole **MxB** dye had a higher dipole moment value (4.57 Debye) and electrophilicity values (20.43 eV) than the **MG** dye (3.19 Debye and 18.36 eV, respectively). Therefore, the **MxB** dye is the most electrophilic dye and consequently will be adsorbed first.

**Table 3:** Total energy, energy of HOMO, LUMO, energy gap, dipole moment, the ionization potential (I, eV), electron affinity (A, eV), chemical hardness (η, eV), global softness (S, eV^−1^), chemical potential (V, eV^−1^), electronegativity (χ, eV), and global electrophilicity index (ω, eV) of **MG-**Al_2_O_3_, **MxB-**Al_2_O_3_ and **MG-**Al_2_O_3_**-MxB** computed at B3LYP/6-31G (d,P) level of theory.

Parameters	MG^24^	MG-Al_2_O_3_	MxB-Al_2_O_3_	MG-Al_2_O_3_-MxB
E_T_ (a.u)	− 1000.84	− 2712.54	− 3717.68	− 3216.97
E_HOMO_ (a.u)	− 8.19618	− 3.17	− 2.79	− 3.68
E_LUMO_ (a.u)	− 5.60374	− 2.82	− 2.19	− 2.90
Energy gap = (E_HOMO_ − E_LUMO)_ eV	2.59	0.35	0.59	0.78
B.E (a.u.) = E_MG-Al2O3-MxB_ − (E_Al2O3-MG_ + E_Al2O3-MxB_ (= − 3216.967 – (− 2712.543/2 + − 3717.678/2) = − 1.86				
Dipole moment μ (debye)	3.190	10.21	18.43	4.97
Ionization potential (I = − E_HOMO_) eV	8.20	3.17	2.79	3.68
Electron affinity (A = − E_LUMO_) eV	5.60	2.82	2.19	2.90
Electronegativity χ = (I + A)/2 eV	6.90	2.99	2.49	3.29
Chemical potential p = − χ eV	− 6.90	− 2.99	− 2.49	− 3.29
Chemical hardness (η = (I − A)/2) ev	1.30	0.17	0.30	0.39
Chemical softness (S = 1/2η) eV	0.39	2.87	1.68	1.28
Electrophilicity index ω = p^2^/2η eV	18.36	25.75	10.43	13.87

From the theoretical study and calculated parameters, we can propose the theoretical interaction between the mixed cationic dyes **MxB** and **MG** with alumina. As shown from the structure of cationic **MxB** (Fig. 1), it has an appositively charged N atom and the cationic **MG** also has an appositively charged N atom on its skeleton. On the other hand, alumina possesses two negative oxygen atoms, therefore, in this system; electrostatic forces are generated when the **MG** and **MxB** ratio (1:1) exist with alumina. So, we can say the interaction occurs due to the attraction force between the positive center (–N-atom) of one (**MG**) molecule with one negatively charged oxygen atom in alumina, and the second negative oxygen atom in alumina attracts the positive –N-atom of (**MxB**) dye (one molecule). So, this interaction has been assigned to electrostatic forces between the cationic groups (–N^+^) of **MxB** and **MG** (equal ratio) and the negatively charged groups (-Al-O^-^) on the alumina surface and a single bond is formed between the negative oxygen atoms and positive nitrogen atoms in the system (Fig. 2). The optimized bond length as shown in Table S2, N–O between **MxB** (N) and the Al-oxide (O), N–O between **MG** (N) and the Al-oxide is 1.38606 Å (literature experimental N–O is 1.1–1.36 Å^31^). It is clear that the increase in the N–O bond between **MxB** and alumina surface, **MG** and alumina surface decreased the strength of the interaction, so, physisorption process was confirmed for the mixture of cationic dyes as occurs in the individual dyes and this is a good point in the adsorption as the presence of other dyes doesn’t impact on the efficiency of adsorption. This result may be related to the excellent surface we use for the adsorption and conciliation in selection of adsorbent.

Moreover, in order to construe the stability of the adsorbed dye molecules at various adsorption sites, the adsorption energy *E*_*ads*_ (Binding Energy) of each model is calculated according to the following formula:

3 $${\text{E}}_{{{\text{ads}}}} = {\text{ E}}_{{{\text{total}}}} {-} \, \left( {{\text{E}}_{{\text{adsorbent \, surface}}} + {\text{ E}}_{{\text{dye \, molecule}}} } \right)$$

where E_total_, E _adsorbent surface_, and E _dye molecule_ are the total energies of the adsorbed system, the clean Al_2_O_3_ surface, and the isolated dye molecule before adsorption, respectively. Accordingly, the stability of the adsorbed molecules after adsorption will be examined based on the positive or negative adsorption energy. In other words, if the adsorption energy is negative, the adsorption process releases heat, indicating that the adsorbed molecule is more stable after adsorption, and vice versa. Consequently, the adsorption energy of those sole and mixed dye molecules onto Al_2_O_3_ was calculated through B.E. Equation (3) ^33^ (c.f. Tables 1 and 3). The obtained results demonstrated that the negative adsorption energy for the binary system (− 1.86 a u) reflected that the adsorbed binary system is energetically more favorable compared to the adsorbed single dye system. So, this result represented that the stability of the adsorbed molecules can be arranged as the following: (Al_2_O_3_-**MxB**) < (**MG**-Al_2_O_3_) < (**MG**-Al_2_O_3_-**MxB**).

### Morphology of alumina sample

**Figure 3:**
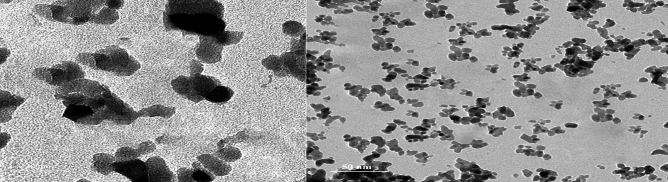
Transmission electron microscopy (TEM) pictures of γ-Al_2_O_3_.

TEM analysis was applied to confirm the actual particle size of alumina which is at the nanoscale (6.25 nm), applicable to X-ray data (4.1 nm)^24^ and represented spherical pores shape as shown in Fig. 3. Also, TEM picture showed very good dispersion and less agglomeration, which will expect to enhance the dye adsorption process.

### Experimental study for impact of different factors on MxB dye removal

#### Contact time and pH of MxB dye solution

**Figure 4:**
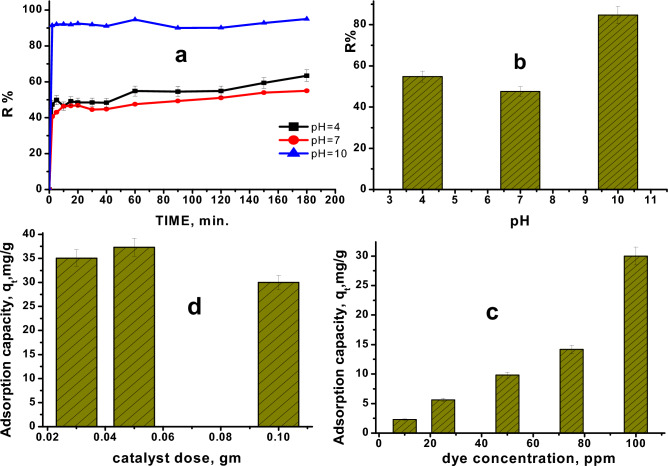
Impact of (**a**) contact time of (**MxB**) dye solution with different pH, (**b**) pH of (**MxB**) dye at equilibrium time on its removal % (adsorbent dose 0.1 g, room temp., initial dye conc. 100 mg/L), (**c**)concentration of (**MxB**) dye solution (adsorbent dose 0.1 g at room temp., pH 10, and at equilibrium time), and (**d**) adsorbent dose (pH 10, room temp., initial dye conc. 100 mg/L, and at equilibrium contact time), on the adsorption capacity of **MxB** dye.

To apply this adsorption process in an industrial ambience, the minimum contact time is preferable for cleaning the dye solution with a high degree of purity. So, the impact of contact time on the adsorption of **MxB** onto the applied adsorbent at various pH values of **MxB** solutions is shown in Fig. 4a. The figure represented that the elimination of the **MxB** dye increased by passing the contact time to the equilibrium for all studied adsorption experiments at different pH values for the dye solution. Because of the availability of more vacant adsorption sites and the easiest penetration of **MxB** molecules into the mesopores of the adsorbent; the adsorption process of the **MxB** onto alumina adsorbent was rapid at the premier stages of the contact duration. Then, as the contact time enhanced, the adsorption sites became less available and a slower adsorption phase was reached until equilibrium after only 20 min.

Figure 4b illustrated the study of the **MxB** solution's adsorption behavior on ɤ-alumina at pH values of 4 ("acid media"), 7 ("neutral"), and 10 ("an alkaline medium"). The results showed that the pH of the dye solution had a significant impact on the adsorption process; the percent of purification of the solution ranged from a low of 49 percent at pH 4 to a maximum of 92.1 percent at pH 10. By going back to the pH_zpc_ of the adsorbent, as shown in Fig. 4b, it is possible to examine the high removal of **MxB** dye at high pH. As known, the pH_zpc_ influences the extent of zero charge on a solid surface in the lack of specific sorption, which is carried out using the powder addition method^34^. Alumina's pH_zpc_ was found to be 7.5, while **MxB**, a cationic dye releases positive ions when dissolved in water. The surface of the adsorbent becomes negatively charged at pH > pH_zpc_, favoring the adsorption of cationic dye owing to an increase in the electrostatic force of attraction. As a result, cation adsorption on ɤ-alumina will be advantageous at this pH level. The opposite, however, occurs at low pH (< pH_zpc_); the positively charged adsorbent surface will repel the positively charged **MxB** cations to generate unoccupied adsorption sites, which reduces dye sorption. Hence, the ion diffusion acts as physical forces which impact on the behavior of the adsorbate molecules in the nearness area of the adsorbent ‘ɤ-alumina’ surface.

However, we can conclude that for the next experiments, the optimum pH and equilibrium time will be achieved at 10 and 20 min, respectively.

#### Maxilon Blue concentration

Figure 4c shows the relationship between the adsorption capacity of **MxB** dye on ɤ-Al_2_O_3_ at equilibrium time (room temperature = 293 K) and dye concentration varying between 10 and 100 ppm. It was found that the percentage of dye adsorbed rose when the dye concentration increased. This might be the result of unsaturated sites existing on the surface of ɤ-alumina. In order to check all of the **MxB** adsorption experiments below, it is preferable to achieve 100 ppm **MxB** concentration in the experiments that follow.

#### Adsorbent amount

The efficiency of the adsorption capacity of **MxB** on γ-Alumina was investigated at optimum conditions by changing the adsorbent dose (0.03–0.1 g), and the results are offered in Fig. 4d. According to this figure, the value of adsorption capacity increased until the adsorbent dose reached 0.05 g. But augmentation of the adsorbent dose led to decreasing the dye adsorption capacity and this result may be associated with an agglomeration and crowding of a greater amount of adsorbents, which block the pores and of course the adsorption process will decrease.

### Experimental adsorption isotherm study

**Figure 5:**
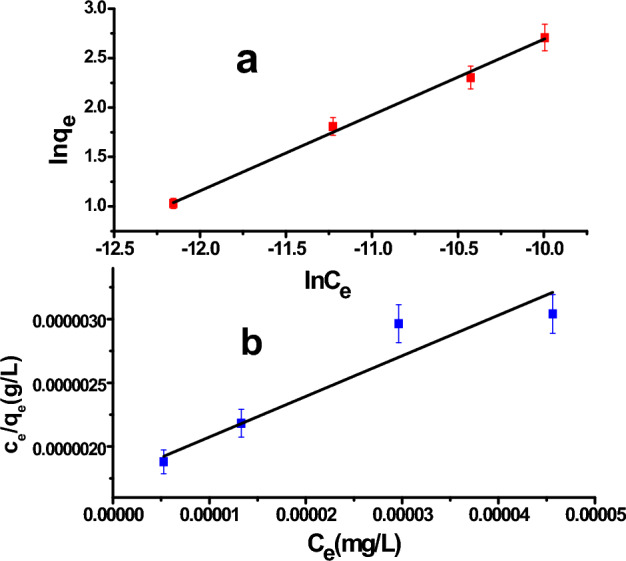
(**a**) Freundlich isotherm model, and (**b**) Langmuir isotherm model of **MxB** dye adsorption on ɤ-Al_2_O_3_ at room temperature.

The retention or release of a material from the aqueous phase to the solid phase at a constant temperature is shown by the adsorption isotherm, as is well know. Understanding the properties of the adsorption surface as well as the mechanism of interaction between the adsorbate and the adsorbent surface is crucial^35^. In the present work, two isotherm models were studied, namely the Langmuir and Freundlich isotherm models, to obtain the best of equilibrium curves^36^. The mathematical equations of the selected isotherm models and their parameter description^37–40^ are listed in Table 4; four calculated model constants and six statistical parameters obtained from the two selected isotherm models are also summarized in Table 4, and the experimental results are shown in Fig. 5a and b.

**Table 4:** Estimated isotherm models and statistical parameters for **MxB** dye adsorption on ɤ-Al_2_O_3_.

Models	Langmuir* $\frac{\text{Ce}}{\textrm{ qe}}=\frac{1}{\text{Kl Qm}}+\frac{\text{Ce}}{\textrm{Qm}}$		Freundlich* $\text{lnqe}=\text{ lnKf}+\frac{1}{\text{n }}\text{ lnCe}$	
Parameters	Q_m_ (mg/g)	K_l_ (L/mg)	K_f_ (mg/g)	1/n
	31.37	18.16 × 10^3^	31.79 × 10^3^	0.76
Adj-R^2^	0.89		0.99	
F-value	26.17		604.39	
Sum of squares	60.31		222.29	
Mean squares	60.30		222.29	
Prob > f	0.036		0.002	
DF	2		2

As is common knowledge, the Langmuir isotherm postulates that once an adsorbate has occupied a spot, no more adsorption occurs there, creating a discriminating plateau in the curve, and there is no side commerce or steric interference between molecules that have been adsorbed^38,41^. On the other hand, the Freundlich isotherm could be considered an empirical model in which there is an interaction between adsorbed molecules (multilayer adsorption) on heterogeneous surfaces with a uniform energy distribution^40,42^. Also, this model proved that the (adsorbate) dye concentration on the adsorbent surface will be increased if there is a growing of adsorbate concentration in the solution without attaining saturation^43^. According to the obtained results in Table 4, adj. R^2^ values revealed a strong linear connection (0.995 close to 1) between the Freundlich model predicted and the experimental values, which is over and above assured by lower values of other statistical parameters such as the probability factor. But the reverse was got by carrying out the Langmuir model. Hence, these results reflected that the experimental values of **MxB** dye adsorption have a stronger correlation with the Freundlich isotherm than Langmuir model. Further, adsorption is a favorable physical process because of the appearance of 1/n parameter in the range of 0–1^36,44^. As well, the q_m_ value was calculated by applying the Langmuir equation, and it was equal to 31.37 mg/g.

Where: *^20,45,46^, q_e_ (mg/g): equilibrium adsorption capacity, q_m_ (mg/g): maximum adsorption capacity, K_l_ (L/mg): Langmuir constant, C_e_ (mg/L): equilibrium adsorbate concentration in solution, K_F_ (mg/g): Freundlich constant, n: Heterogeneity factor.

### Adsorption kinetic study of single Maxilon Blue dye

It is necessary to understand the kinetics and mechanism of adsorption in order to establish a good water treatment system. As liquid solution is adsorbed onto the solid adsorbent surfaces in many phases, the slowest step actually controls the entire process. Simulations of the linear form of the pseudo-first order (PFO) were carried out using data from the adsorption kinetic studies for different dye concentration solutions, pseudo-second order (PSO), and Elovich models to look into the sorption process of **MxB** dye onto ɤ-alumina adsorbent. Their equation forms are listed below Table 5.

**Table 5:** Kinetic and statistical parameters of **MxB** dye adsorption onto ɤ-Al_2_O_3_ at different dye concentrations (10–100 mg/L).

Kinetic model	Parameters			Sum of squares	Mean square	F-value	DF	P-value	
	K_1_ (min^−1^)	q_e_ (mg/g)	Adj-R^2^						
PFO*									
10	0.001	0.94	0.69	4.38	4.38	27.64	11	2.70 × 10^–4^	
25	0.026	2.48	0.90	18.50	18.50	97.88	10	1.75 × 10^–6^	
50	0.030	1.03	0.96	40.68	40.68	281.98	11	3.46 × 10^–9^	
75	0.029	2.91	0.78	36.17	36.17	44.16	11	3.63 × 10^–5^	
100	0.025	8.55	0.93	26.54	26.54	155.84	11	7.74 × 10^–8^	

Kinetic model	Parameters				Sum of squares	Mean square	F-value	DF	P-value
	q_e(exp.)_ (mg/g)	K_2_ (Conc^−1^ min^−1^)	q_e_ (mg/g)	Adj-R^2^					
PSO**									
10	2.8	0.1	2.57	0.99	6679.77	6679.77	1333.26	11	7.83 × 10^–13^
25	6.1	0.03	6.24	0.99	704.17	704.17	1511.98	10	3.02 × 10^–12^
50	10	0.13	10.03	0.99	439.38	439.38	1.78 × 10^5^	11	0
75	15	0.04	14.91	0.99	198.54	198.54	1.12 × 10^4^	11	0
100	31.7	0.01	31.76	0.99	43.78	43.78	1.88 × 10^4^	11	0

Kinetic model	Parameters			Sum of squares	Mean square	F-value	DF	P-value	
	α (mg/g min)	β (g/mg)	Adj-R^2^						
Elovich***									
10	8.65	3.12	0.61	3.35	3.35	19.61	11	0.001	
25	5.18	1.08	0.79	24.31	24.31	43.09	10	6.35 × 10^–5^	
50	176.29	0.93	0.39	37.91	37.91	8.54	11	0.0139	
75	166.90	0.60	0.44	89.51	89.51	10.40	11	0.008	
100	113.06	0.25	0.61	524.34	524.34	19.54	11	0.001	

*ln (q_e_ − q_t_) = ln(q_e_) – k_1_t^34,44^, **$\frac{\text{t}}{\textrm{qt}}=\frac{1}{k2 qe}+\frac{\text{t}}{\textrm{qt}}$ ^44,45,48^,*** q_t_ = $\frac{1}{\beta }$ ln (α β) +$\frac{1}{\beta }$ ln t^33^, where, q_t_ is the amount of dye adsorbed at time t (mg/g), k_1_ is the first order rate constant (min^−1^), k_2_ is the pseudo-second order rate constant (conc. min)^−1^, α (mg/g min) is the initial sorption rate, and the parameter β (g/mg) is related to the extent of surface coverage.

**Figure 6:**
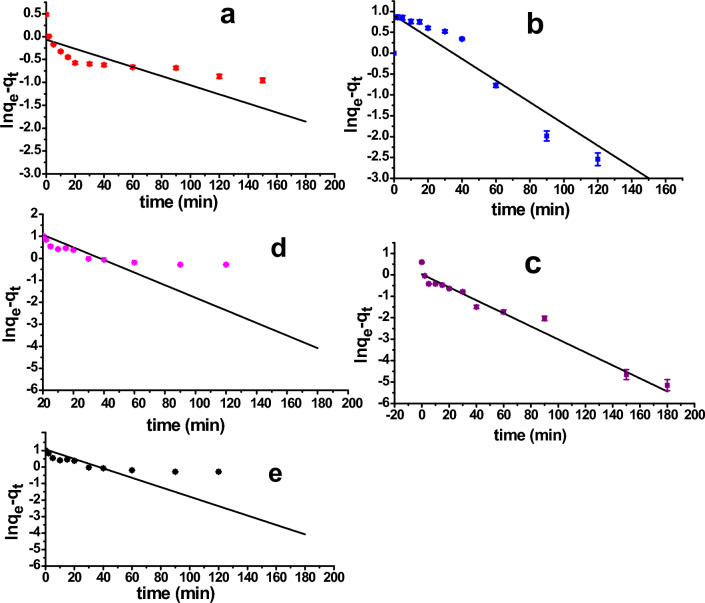
Linear form of PFO of **MxB** dye adsorption on ɤ-Al_2_O_3_ at room temperature for different dye concentrations (10, 25, 50, 75, and 100 mg/L) (**a**–**e**), respectively.

**Figure 7:**
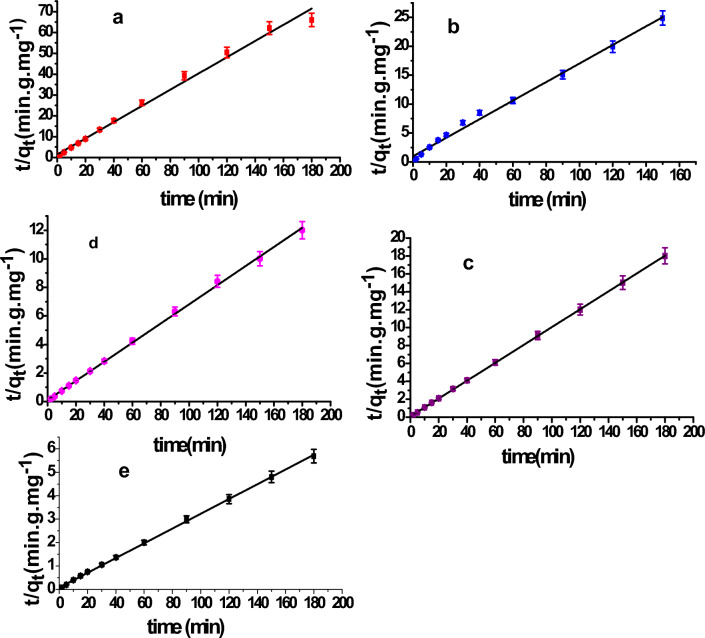
Linear form of PSO of adsorption of **MxB** dye on ɤ-Al_2_O_3_ at room temperature for different concentrations (10, 25, 50, 75, and 100 mg/L) for (**a**–**e**), respectively.

As in Fig. 6a–e, the linearity plots of ln (q_e_–q_t_) versus time “t” for the pseudo-first order (PFO) model were obtained. The kinetic parameter values of k_1_, q_e_, the adj. correlation coefficient (R^2^) values, and the statistical parameters of fitting the (PFO) for **MxB** dye adsorption are listed in Table 5. From Table 5, it is clear that the lower value of adj. R^2^ and the higher value of statistical parameters indicated that the adsorption process did not obey the (PFO) model. On the other side, the parameters of linear forms of (PSO) equations emphasized that the rate-limiting step resulted from the chemical interaction between the solute and the adsorption sites at the adsorbent surface. To understand the achievement of this model, linear plots of t/q_t_ versus time “t” for different concentrations were shown in Fig. 7a–e. The k_2_, q_e_, adj-R^2^, and statistical parameters were calculated from the plots and recorded in Table 5. As shown in this table, the theoretical adsorption capacity q_e_ determined from the (PSO) model is nearby to the practical adsorption capacity q_exp_. Furthermore, the adj. R^2^ is also greater than 0.99 (∼ 1), and statistical parameters have the lowest value (prob. value = 0). We can definitely say that the pseudo-second-order model could well describe the system.

Elovich kinetic model in linear form showed that a linear relationship was obtained between q_t_ and ln t over the whole adsorption period as shown in supplementary data (see Fig. S1a–e) and Elovich parameters were recorded in Table 5. These parameters are associated with the activation energy for the chemisorption process and are helpful in describing the adsorption on highly heterogeneous adsorbents as alumina, which possesses heterogeneous surface-active sites^47^.

### Adsorption of mixture of MxB and MG dyes

**Figure 8:**
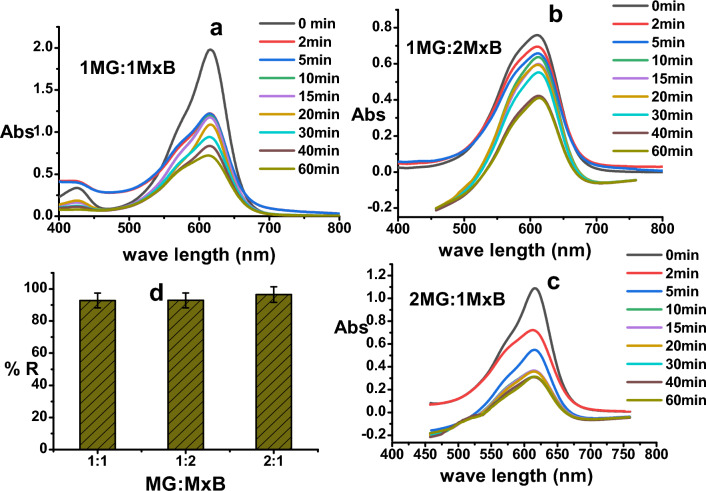
(**a**–**c**) UV–visible Absorbance of a mixture of **MG** and **MxB** with different ratio, and (**d**) the removal % of these mixtures of dyes with different percentages (pH 7, room temp., 0.1 g dose of ɤ-Al_2_O_3_ and dye conc. = 50 mg/L).

For the mixture of two cationic dyes **MG** and **MxB** which were studied at the conditions mentioned earlier, the UV–vis spectra of the different mixtures (**MxB**: **MG**) Fig. 8a–c reflected that a new λ_max_ = 612 nm appeared that is not far away from the λ_max_ of two single dyes (609 nm for **MxB** and 617 nm for **MG**) but in between, which emphasized the equivalent selectivity toward the removal of two dyes in the binary system at equilibrium time as shown in Fig. 8d without forming an intermediate. So far, the removal efficiency of the mixture of **MxB** and **MG** significantly did not change with increasing the percent of each of them. Only a very small increase in the R% didn't exceed 1.8% for **2 MG**: **1 MxB**. This behavior not only revealed the greater affinity for both dyes but also proved the absence of selectivity of the adsorbent toward adsorbing these dyes in the bi-adsorbate system. This might be attributed to the similarity in their cationic nature. Further, the removal percentage of various mixed dyes at the same conditions was close to R% for sole **MxB** and **MG** (~ 93.6, 98.3%, respectively). From these obtained results, it is clear that the prepared adsorbent gave very high decolorization efficiency for a mixture of cationic dyes, showing its qualification for water treatment, especially in binary systems.

### Interaction mechanism of MxB and/or MG mixture dyes with Al_2_O_3_ adsorbent

To the best of our knowledge, the surface behavior of ɤ-alumina, the structure of the dye molecule, and the interaction of the dye with the adsorbent are responsible for the mechanism of the process. The mechanism of dye adsorption through the *electrostatic interaction* was expected to be the main energetic force, which had been confirmed by previous research^48,49^.

In discussion of the interaction mechanism of **MxB** and/or **MG** mixture dyes with Al_2_O_3,_ initially, the theoretical results obtained by DFT calculations can be concluded in the following:

1. The sole or binary dye adsorption mechanism occurred through *electrostatic interaction* because of the creation of an electrostatic interaction between the (N^+^) ion of **MxB** or **MG** and (O^−2^) on the alumina surface. So, this interaction occurred between two **MxB** and/or **MG** dye molecules and one alumina molecule.
2. The optimized bond length N–O between dye and oxide adsorbent is larger than the literature, which supports the *physisorption process* for the adsorption of single or binary cationic dye systems.
3. The similarity of many theoretical reactivity descriptor parameters for **MxB** and **MG** proved that there was *no preferable dye* in the mixed binary system for adsorbing over alumina.
4. The negative adsorption energy data of those sole and mixed dye molecules onto Al_2_O_3_ are arranged as follows: (Al_2_O_3_-**MxB**) < (**MG**-Al_2_O_3_) < (**MG**-Al_2_O_3_-**MxB**), which referred that the **MxB** dye is the less stable dye to be adsorbed.
5. According to the previous outcome, energetically, the adsorbed *binary system is more favorable* than the adsorbed sole dye.

**Scheme 1:**
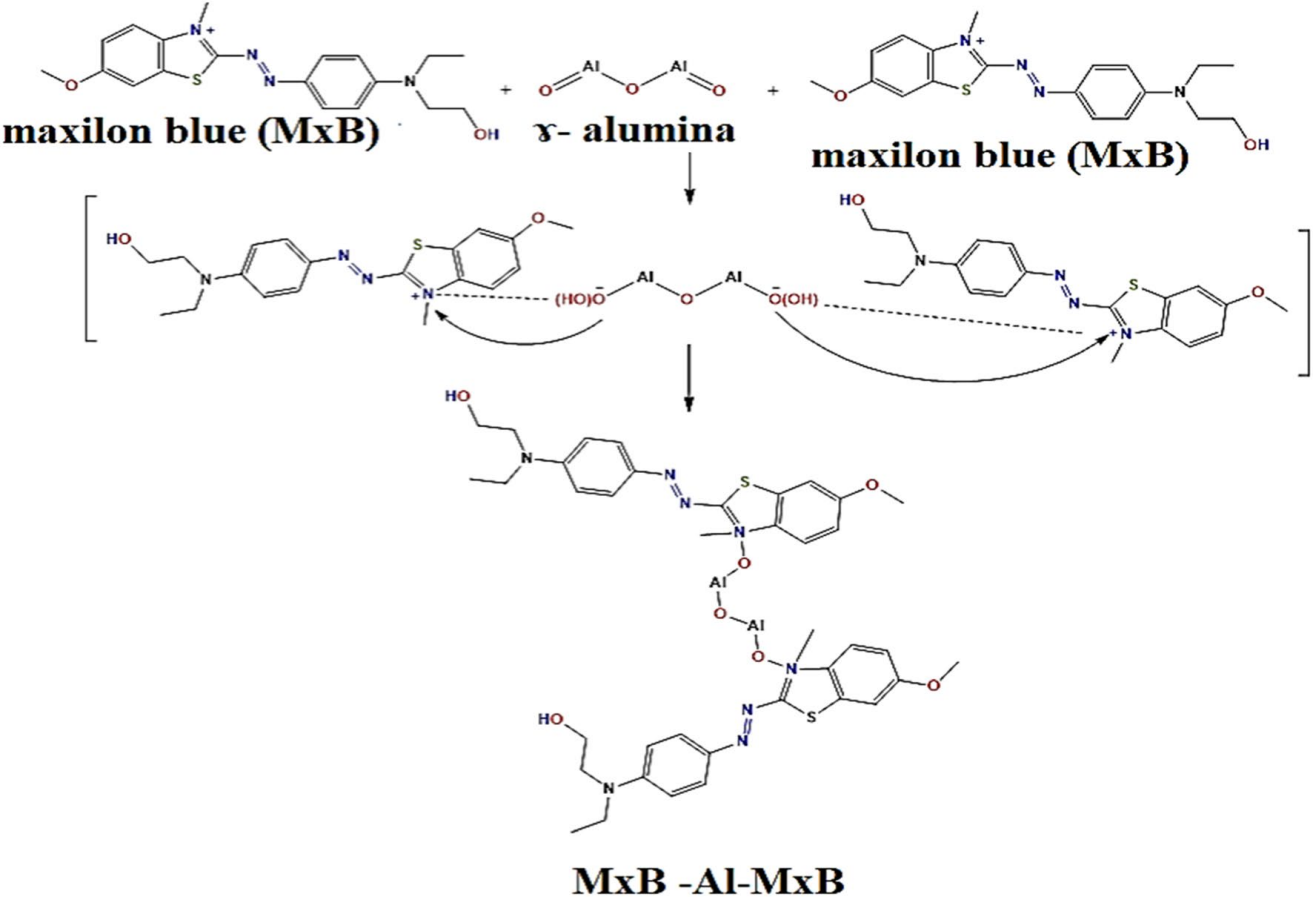
Schematic illustration of the interaction mechanism of the sole dye adsorption process onto Al_2_O_3_ adsorbent.

**Scheme 2:**
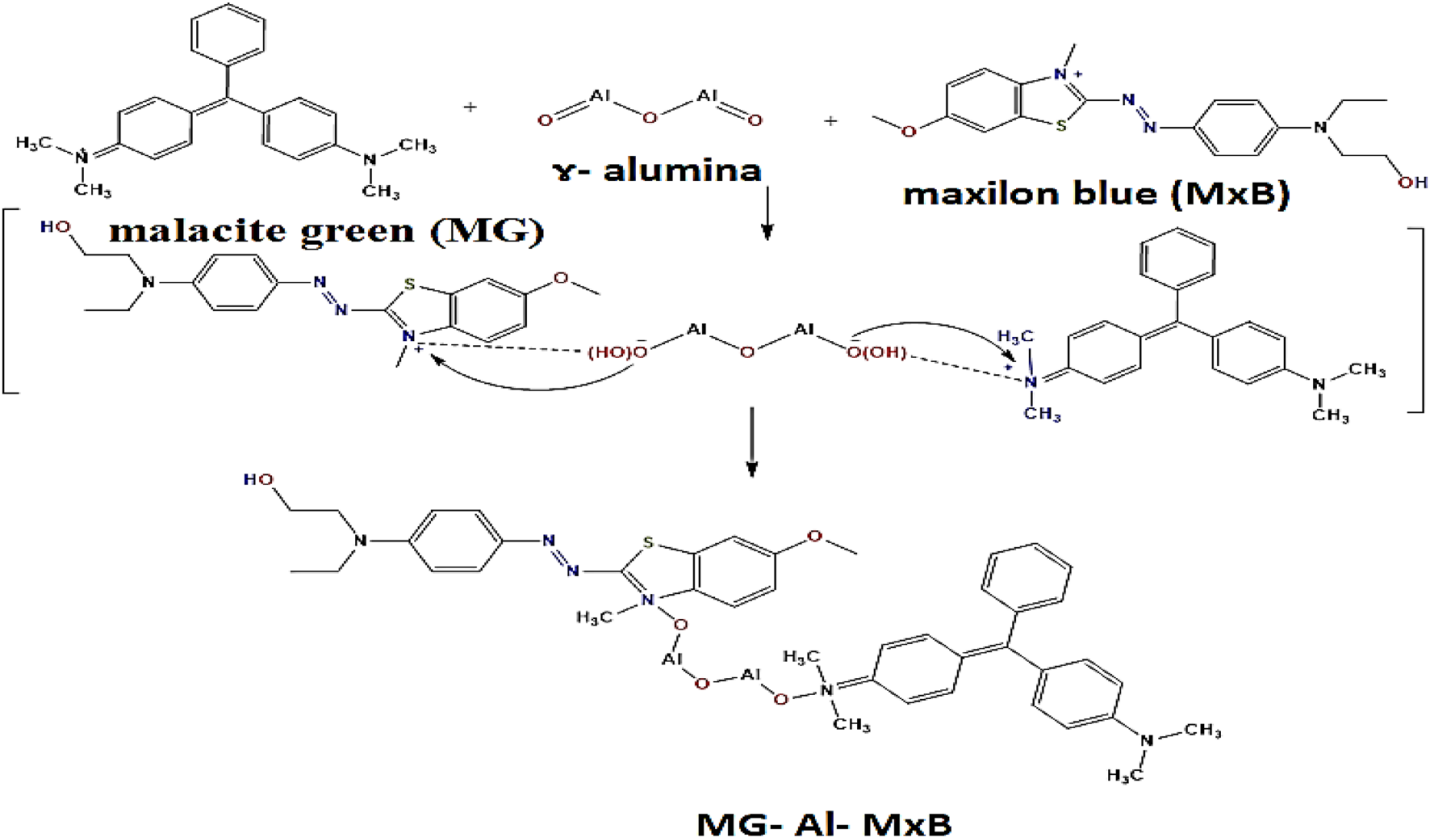
Schematic illustration of the interaction mechanism of the binary dye adsorption process onto Al_2_O_3_ adsorbent.

On the other side the experimental results revealed the following:

1. The low-cost alumina adsorbent can be used as a highly efficient adsorbent for cationic dyes from an aqueous solution due to its advantages of small nanometer scale, high S_BET_, high porosity, and good dispersion.
2. The **MxB** cationic dye releases positive ions when dissolved in water, while the adsorbent surface becomes negatively charged at high pH (> pH_zpc_), favoring the occurrence of *electrostatic interactions* between the positively charged nitrogen and the negative oxygen center (Scheme 1).
3. The behavior of the dye molecules in the vicinity of the adsorbent "ɤ-alumina" surface is affected by the physical force of ion diffusion.
4. The experimental data of **MxB** dye adsorption represented a stronger correlation with the Freundlich isotherm, so, the *physical mechanism of adsorption* is a favorable process.
5. Kinetically, the pseudo-second-order model could well describe the system.
6. Further, the adsorption abilities toward the binary system (**MxB** and **MG**) were favorable owing to electrostatic attractive forces between the more positively active centers in two cationic dyes and the more negatively charged centers in the alumina surface with the physisorption process (Scheme 2).
7. The adsorbent's *lack of selectivity* in the bi-adsorbate system for adsorbing **MG** and **MxB** dyes.

From the previous integrated results, it is clear the good agreement between the experimental and theoretical outcomes.

**Figure 9:**
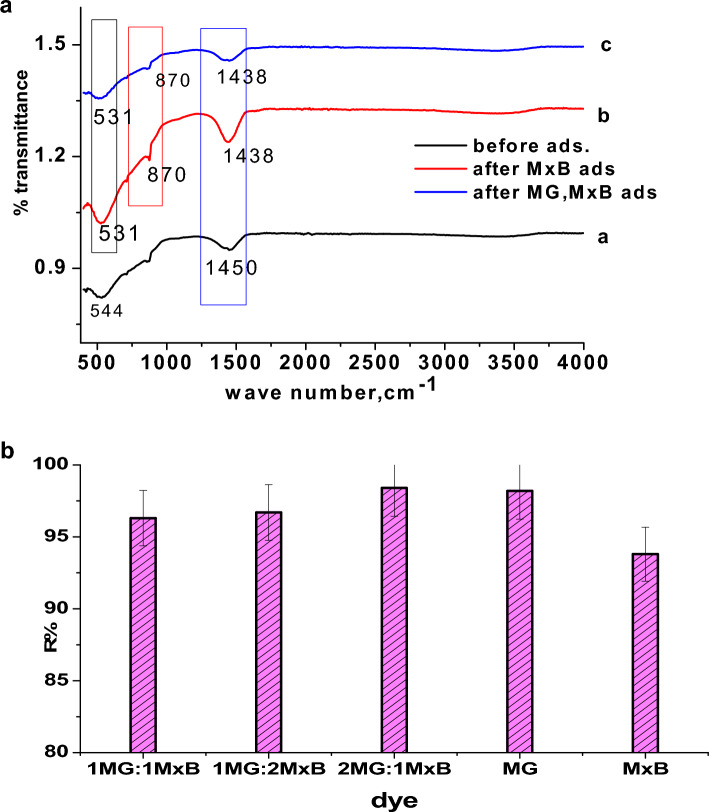
(**a**) FT-IR spectrum of (a) adsorbent before dye adsorption, (b) after **MxB** dye adsorption, and (c) after **MxB** and **MG** mixture dye adsorption. (**b**) Comparison the removal efficiency % for sole and mixture of cationic dyes with different percentages (pH 7, room temp., at equilibrium time, 0.1 g dose of Al_2_O_3_ and dye conc. = 50 mg/L**).**

In order to extra analyze the adsorption mechanism of dyes onto alumina, the FTIR spectra of alumina are shown in Fig. 9a. In comparison with adsorbent before and after adsorbing different dyes, certain changes were detected in the FTIR spectra, at which the peak at 1450 cm^−1^ was attributed to the non-bridging of O–H transformed into 1438 cm^−1^ in Al_2_O_3_-**MxB** and **MG**-Al_2_O_3_-**MxB**. In addition to the appearance of the bands at 870 and 531 cm^−1^ which are assigned to some interaction among Al (III) and oxygen or hydroxide bridge^50^. Based on the above-mentioned changes in FTIR spectra, it can be deduced that the adsorption mechanism of single **MxB** or its doublet with **MG** dye adsorption may occur through *hydrogen bonding* between the hydrogen of –OH groups on the surface of alumina and the nitrogen in the dye molecules for adsorbing **MxB / MG** (Schemes 1–2). Further, from the arrangements of **MG** and **MxB** over Al_2_O_3_ in the binary system (Fig. 2a,b), we noticed that the dimethyl amino group in **MG** revealed a tilt in the aromatic ring; however, for **MxB**, the entire aromatic ring is aligned over the adsorbent. So, the adsorption process can be expected to occur through the dispersion interaction, firstly for **MxB** over alumina with the physisorption process.

Figure 9b showed that at the end of the adsorption process, a comparison of the decolorization efficiency onto alumina adsorbent for different single and double cationic dyes at equilibrium time was the lowest for **MxB** over Al_2_O_3_, and this result may be due to its low stability as shown previously from the adsorption energy values (Table 3).

Thermodynamic behavior of the adsorbent solid toward **MG** adsorption was experimentally studied in our previous work^24^, which indicated that the adsorption process for cationic dye is spontaneous, endothermic in nature and increase the entropy occurred reflecting an increase in the randomness near the solid/solution interface. Logically, the adsorption behavior of **MxB** dye thermodynamically will be the same as the **MG** dye adsorption.

Finally, the last information can be summarized: the dye adsorption mechanism occurred through *electrostatic interaction* and/or *hydrogen bonding* through the physisorption process. The binary adsorption system exhibited the higher stability of the complex (**MG**-Al_2_O_3_-**MxB**) on alumina than other complexes.

### Regeneration studies

A critical factor in determining commercial viability is the efficiency of an adsorbent to be reused. In this section, the used adsorbent was washed several times with distilled water, vacuum-dried at 110 °C, and then utilized again in subsequent cycles for adsorption. Generally, the % R over Al-2 adsorbent for 4 cycles of MxB dye adsorption was investigated as in Fig. S2 "see supplementary data". The obtained results revealed that the % R over the regenerated adsorbent was slightly decreased at the fourth cycle, but it could still represent around 70%. The stability of the Al-2 adsorbent after reusing it for the **MxB** adsorption process has been investigated by XRD, as shown in supplementary data (see Fig. S3). According to the XRD study, the Al-2 after regeneration did not significantly alter peak locations or powder size. This result indicated that the prepared adsorbent has significant stability even after severally uses and certain adsorbent mass may be consumed with repeated use. Further, the prepared adsorbent efficiency toward cationic dyes was the highest compared with other previously studied adsorbent results, as shown in Table 6^22,24,48,51–60^. Finally, we can conclude that the nano-adsorbent demonstrated excellent stability in both theoretical and experimental results compared with previous studies^61–66^.

**Table 6:** Comparison of cationic dyes removal efficiency using different materials via adsorption.

No.	Adsorbent	Cationic dye	Adsorption condition				Efficiency (%)	Ref.
			Dye conc	Adsorbent dose, mg	Time, min	Temp., °C		
1	CNC	MB	–	100	180	RT	78	^54^
2	MCMFCs		20 ppm	10	150	–	92	^55^
3	CMC/GOCOOH		20 ppm	50	300	RT	95	^56^
4	HPAM/CNC		5 ppm	2	240	–	90	^57^
5	CMC coated Fe_3_O_4_@SiO_2_ MNPs		50 ppm	50	720	–	85	^58^
6	CoFe_2_O_4_/H6 300 °C		100 ppm	100	120	RT	40	^48^
7	CoFe_2_O_4_/H8 300 °C		100 ppm	100	120	RT	50	^48^
8	Chitosan beads	MG	40ppm	25	300	–	88	^59^
9	ZIF-8		50 ppm	62	240	–	80	^51^
10	MgO nano-rod		50 ppm	10	1500	–	92	^52^
11	Al-2		50 ppm	100	20	25	98.8	^24^
12	Sepiolite	MxB	2.5 × 10^−3^ M	5 × 10^3^	180	25	85	^60^
13	Al-2		100 ppm	50	20	20	99	Present study
14	Nano-alumina	Rhodamine blue	10^–6^ M	30	180	–	80	^22^
15	Activated carbon from apple leaves "AC1"	Basic dye C.I. base blue 47	60 ppm	100	150	25	50.4	^53^

### Conclusion

An innovative study for the adsorption of single (**MxB**) and its mixture with (MG) cationic dye from aqueous solution in different ratios was carried out on γ-alumina experimentally and simulated theoretically by DFT theory. Theoretically, the full geometry optimization of **MxB** and its mixture with MG dyes with a ratio of 1:1 was performed to investigate the structure geometry and energetics by applying the B3LYP/6-31G (d, p) level of theory. There was a good agreement between the theoretical and the experimental results, as the following: (i) the optimized bond length N–O between **N-atom** of the dye and the (O) atom of Al-oxide was higher than the literature. This result confirmed the physical adsorption process in the sole or binary systems. (ii) Studying the non-linear optical properties of the formed complexes after adsorption showed that the complex **MG**-Al_2_O_3_-**MxB** is promising as an NLO material and can be used in photo- applications. (iii) Also, a comparison of the removal efficiency for the single and the mixture of cationic dyes was performed experimentally and theoretically. The outcomes referred that the adsorption of the mixture of binary dyes on alumina is more preferable than the single system due to its high stability. But, the similarity of some theoretical reactivity descriptor values for **MxB** and **MG,** and their experimental adsorption results reflected that there was no better dye in the mixed binary system for adsorbing on alumina. (iv) Further, the interaction mechanism for **MxB** and/or **MG** was proved and may be due to the electrostatic interaction between the cationic groups (–N^+^) of **MxB** and/or **MG** (equal ratio) and the negatively charged groups (–Al–O^−^) on the alumina surface or through hydrogen bonding.

Some experimental items were studied on the adsorption of sole dye onto nanoadsorbent for optimization. It was revealed that the **MxB** removal reached to 99% at ideal conditions (pH 10, dye concentration = 100 mg/L, adsorbent dose = 50 mg, and equilibrium time = 20 min). The adsorption of **MxB** was found to obey pseudo- second order model and Freundlich isotherm for adsorption the kinetics and isotherms, respectively. The nano-adsorbent demonstrated excellent stability and reusability across four cycles.

### Supplementary Information

Supplementary Information.

## Data Availability

All data generated or analyzed during this study are included in this published article and its supplementary information files.
