# Supplementary material for: New combined experimental and DFT studies for adsorption of sole Azo-dye or binary cationic dyes from aqueous solution
Source: Sci Rep. 2024 Jun 26;14:14756. doi: 10.1038/s41598-024-65649-2 (PMC11208540; doi:10.1038/s41598-024-65649-2)
Supplement: Supplementary file 1 — Supplementary Information. [file 41598_2024_65649_MOESM1_ESM.docx]

**Table S1**: Geometrical optimization of **MxB**, and natural charges which computed at B3LYP/6-31G (d,p)level of theory.

| **Maxilon blue (GRL)** | **Parameters** |
| --- | --- |
| **Bond length (A^O^)** | |
| 1.35 | C3-O7 |
| 1.41 | C2- C3 |
| 1.08 | C2-H12 |
| 1.39 | C1-C4 |
| 1.40 | C4-N15 |
| 1.47 | N15-C20 |
| 1.34 | C17-N18 |
| 1.31 | N18-N19 |
| 1.35 | N19-C29 |
| 1.43 | C25-C26 |
| 1.36 | C26-N34 |
| 1.47 | N34-C37 |
| 1.53 | C37-C38 |
| 1.42 | C38-O39 |
| 1.76 | C5-S16 |
| 1.76 | S16-C17 |
| **Bond angle(^O^)** | |
| 105.60 | <H10-C8-O7> |
| 119.31 | <C8-O7-C3> |
| 115.19 | <O7-C3-C6> |
| 119.12 | <C3-C6-H14> |
| 127.72 | <C6-C5-S16> |
| 89.79 | <C5-S16-C17> |
| 112.72 | <S16-C17-N15> |
| 125.32 | <S16-C17-N18> |
| 111.40 | <C17-N18-N19> |
| 117.34 | <N18-N19-C29> |
| 31.05 | <C24-C29-C28> |
| 121.60 | <C26-N34-C35> |
| 31.88 | <C37-N34-C35> |
| 30.16 | <N34-C37-H47> |
| 107.94 | <C37-C38-O39> |
| **Dihedral angle(^O^)** | |
| -179.90 | <H10-C8-O7-C3> |
| 179.89 | <C8-O7-C3-C6> |
| -179.97 | <O7-C3-C6-C5> |
| 179.94 | <C3-C6-C5-S16> |
| -179.94 | <C6-C5-S16-C17> |
| 179.88 | <C5-S16-C17-N18> |
| 0.10 | <N15-C17-N18-N19> |
| -179.61 | <C17-N18-N19-C29> |
| -179.51 | <N18-N19-C29-C24> |
| 170.10 | <C25-C26-N34-C35> |
| 175.85 | <H47-C37-C38-O39> |
| -178.68 | <H40-O39-C38-C37> |

**Table S2:** Selected theoretical bond lengths, for **MxB- Al_2_O_3_** computed at B3LYP/6-31G (d, P) level of theory.

| **Parameters** | **MxB-Al_2_O_3_** |
| --- | --- |
| **Bond length (Å)** | |
| C17-N15 | 1.33 |
| N15-O99 | 1.38 |
| O99-Al102 | 1.91 |
| Al102-O100 | 1.91 |
| O100-Al103 | 1.89 |
| Al103-O101 | 1.91 |
| O101-N64 | 1.36 |
| C53-N64 | 1.49 |
| N64-C66 | 1.28 |
| C66-S65 | 1.76 |
| S65-C54 | 1.75 |
| S65-C66 | 1.76 |
| C66-N67 | 1.45 |
| N67-N68 | 1.24 |
| N68-C78 | 1.47 |
| **Bond angle (^0^)** | |
| <N15-O99-Al102> | 135.73 |
| <O99- Al102-O100> | 160.38 |
| <Al102-O100-Al103> | 122.61 |
| <Al103-O101-N64> | 108.44 |
| <C69-N64-C53> | 34.23 |
| <O101-N64-C69> | 108.85 |
| <C69-N64-C66> | 116.20 |
| <C66-N67-N68> | 106.41 |
| <C75-N83-C84> | 110.12 |
| <H89-O88-C87> | 109.59 |
| <C86-N83-C84> | 109.71 |
| **Dihedral angle (^0^)** | |
| <S16-C17-N15-O99> | 37.42 |
| <N19-N18-C17-N15> | 148.81 |
| <C17-N15-O99-C20> | 130.38 |
| <Al102-O100-Al103-O101> | 166.67 |
| <O100-Al103-O101-N64> | 49.06 |
| <Al103-O101-N64-C69> | 67.66 |
| <S65-C66-N67-N68> | 0.38 |
| <C66-N67-N68-C78> | 143.08 |

**Table S3:** Selected theoretical bond lengths, for **MG-Al_2_O_3_-MxB** computed at B3LYP/6-31G (d, P) level of theory.

| Parameters | **MG-Al_2_O_3_-MxB** |
| --- | --- |
| **Bond length (Å)** | |
| Al1-O2 | 1.82 |
| Al1-O55 | 1.70 |
| O55-Al54 | 1.79 |
| Al54-O53 | 1.97 |
| O53-N25 | 1.48 |
| N56-O2 | 1.52 |
| **Bond angle (^0^)** | |
| <C57-N56-O2> | 109.16 |
| <C58-N56-O2> | 35.55 |
| <O2-Al1-O55> | 128.95 |
| <Al1-O55-Al54> | 135.92 |
| < O55-Al54- O53> | 80.70 |
| < Al54- O53-N 25> | 124.29 |
| < O53-N 25-C26> | 101.50 |
| < C27-N 25-C26> | 34.34 |
| <C18-C19-N25> | 118.71 |
| **Dihedral angle (^0^)** | |
| <C57-N56-O2-Al1> | 122.39 |
| <Al54-O53-N25-C27> | 57.64 |
| <O53-N25-C19-C20> | 134.05 |
| <N25-C19-C20-C21> | 175.68 |
| <C18-C19-C20-C21> | 0.51 |

**Fig .S1:** Linear form of Elovich of **MxB** dye adsorption on ɤ- Al_2_O_3_ at room temperature and different concentrations (10, 25, 50, 75, and 100 mg/L) for (a, b, c, d, and e), respectively.

**Fig. S2.** Recyclability of prepared adsorbent for the MxB dye adsorption (pH=10, room temperature, at equilibrium time, 0.05 g dose of adsorbent and dye conc. = 100 mg/L**).**

**Figure S3.** XRD patterns of powder Al-2 before and after MxB dye adsorption and regeneration process.
